# Conservative treatment versus transverse pinning in fifth metacarpal neck fractures in active adults: a randomized controlled trial

**DOI:** 10.1007/s00068-023-02417-3

**Published:** 2023-12-27

**Authors:** Sherif Hamdy Zawam, Begad Hesham Abdelrazek, Aly Elmofty, Ahmed Morsy, Mahmoud Abousayed

**Affiliations:** Department of Trauma and Orthopedics, Faculty of Medicine, Cairo University, Giza, Egypt

**Keywords:** Boxers fracture, Ulnar gutter slab, Transverse pinning, Fifth metacarpal

## Abstract

**Purpose:**

Compare two simple ways for treating boxer’s fractures in active adults; conservative management by ulnar gutter slab and transverse pinning in fixation of fifth metacarpal's neck fracture regarding union, functional outcomes, and complications.

**Patients and methods:**

Ninety patients with fifth metacarpals' neck fractures with palmar angulation (30–70°) were managed either conservatively by an ulnar gutter slab or surgically by transverse pinning technique from January 2020 to December 2021. Only 84 patients completed a 1-year follow-up. Patients with old, open, or mal-rotated fractures were excluded. The block-randomization method was used to create equal groups. Patients were evaluated clinically and radiologically every 2–3 weeks until union, then at 6 and 12 months. Functional assessment at the final visit was done using the quick DASH score, total active motion (TAM), and total Active Flexion (TAF).

**Results:**

The mean radiological union time for the conservative group in this study was 7.76 weeks, while for the transverse pinning group, it was 7.38 weeks. There was no statistically significant difference between the two techniques regarding union rates and functional outcomes. All patients returned to their pre-injury jobs and level of activity.

**Conclusion:**

Both conservative management in ulnar gutter slab and percutaneous transverse pinning are considered effective methods in the treatment of simple extra-articular fifth metacarpal neck fractures with angulation between 30 and 70 degrees (AO: 77 A3.1). The functional and radiological results using both methods were satisfactory and statistically comparable.

## Introduction

**Fig. 1:**
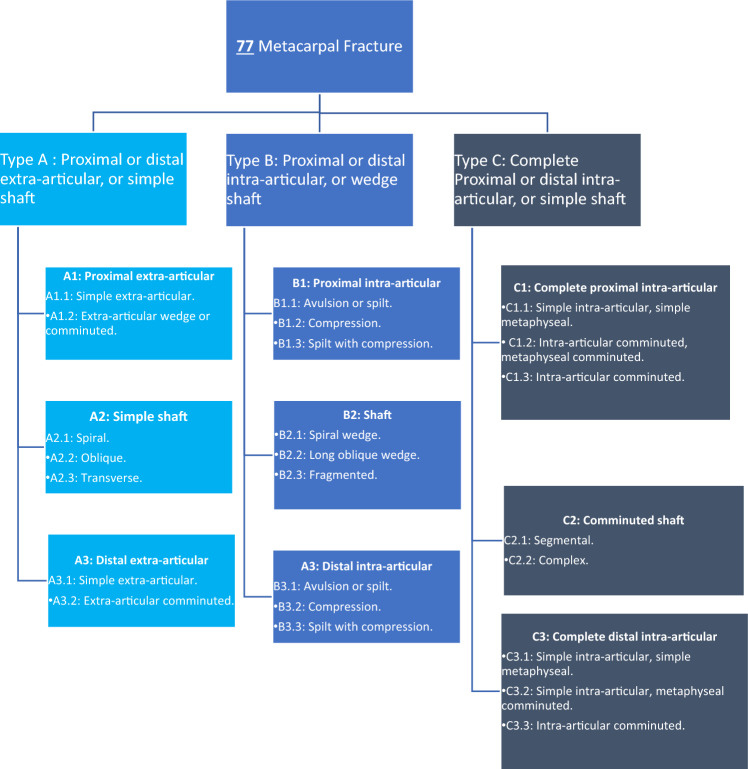
AO classification of metacarpal fractures

Fractures of the fifth metacarpal bone are the most common type of hand fractures [1]. Males are 5 times more affected than females [2]. Fifth metacarpal fractures represent 37% of metacarpal fractures and 18.4% of all hand fractures [3]. Several classifications describe metacarpal fractures such as the well-known classification of the AO (Fig. 1) [4]. Boxer’s fracture usually occurs due to smacking a clenched hand against a hard surface [5].

Despite the high incidence, there is a lack of consensus on the ideal management of boxer’s fracture which represents a challenge. Management includes conservative and operative options. Surgical intervention has absolute indications including open fractures, rotational deformity, irreducible angulation of more than 70 degrees, and multiple metacarpal/phalangeal fractures [6, 7].

1. Conservative treatment: This can be done using Plaster of Paris (POP) slab, functional splints, or even buddy-taping. It is a cost-effective method that requires less time to full recovery. However, it may be associated with loss of reduction, and malunion, especially in the sagittal plane which may cause loss of the normal prominence of the metacarpal head “the knuckle appearance”. Significant shortening, if occurring, will affect the power of the hand grip. Non-union, stiffness, and complex regional pain syndrome (CRPS) are among other potential complications of management in POP [6–9].
2. Surgical treatment: several options exist including k-wires, plates and screws, intramedullary screws, external fixation, and tension band wiring. Surgical treatment aims at achieving good reduction and stable fixation. However, it is more expensive and carries a risk of infection especially if open reduction was performed [10–12].

K-wire pinning has the advantage of being flexible, providing adequate stabilization, preserving the soft-tissue envelope, and is more economical when compared to mini-plates and screws [13]. Intramedullary pins are among the most commonly used techniques. However, it has the disadvantage of requiring a relatively longer operative time than other fixation techniques [1, 14, 15].

Transverse k wiring is an easy technique that provides adequate rotational stability. It involves fixing the fractured metacarpal to an intact adjacent metacarpal [15–17]. 

In this study, we aimed to compare two simple ways of treating boxer’s fractures in active adults; conservative management using an ulnar gutter slab and surgical transverse pinning. The hypothesis is that non-operative treatment using an ulnar gutter splint yields comparable results to transverse pinning of fifth metacarpal neck fractures with equivalent union rates, functional outcomes, and complication rates and that both are valid treatment options.

## Patients and methods

Between January 2020 to December 2021, 90 patients with metacarpal neck fractures who presented at the Emergency department of our tertiary trauma center and met the inclusion criteria were enrolled in this study. Patients were followed up for an average period of 12–14 months. Only 84 patients completed the follow-up, while 6 patients were lost to follow-up. Patients were classified using the AO classification, then the degree of angulation was measured.

We included all patients from 18 to 45 years old, with closed metacarpal neck fractures classified (77 A3.1) as simple distal extra-articular according to the AO classification. The fracture must be recent with a history of trauma of less than 2 weeks, and the palmar angulation ranges from 30 to 70 degrees.

We excluded patients with old fractures (> 2 weeks), and those with less than 30° palmar angulation who were managed conservatively. Furthermore, patients with clear indications for operative management were excluded such as those with open fractures, multiple metacarpal fractures, malrotation, or more than 70° palmar angulation. Ethical approval from our Local Research Ethics Committee was obtained (Institutional Review Board (IRB) number: MS-341-2020.)

The block-randomization method was used to create equal groups. The data were hidden using the closed-envelope method. Ninety closed envelopes were prepared, each had a card numbered from 1 to 90. Every time a patient presented to the ER and was recruited for the trial, an envelope was chosen by the resident on call. The cases were then classified into each group by the odd/even technique. Cards with even numbers were allocated to Group A which included 42 patients, who were managed conservatively using an ulnar gutter slab. Group B (odd numbers) included 42 patients who received operative fixation using closed reduction and transverse pinning using K-wires.

### Ulnar gutter (group A)

The patient was seated or placed in the supine position, the skin was prepped with betadine solution and 3–5 ml of 1% lidocaine was injected into the fracture site. Then, flexion of MCP and PIP to ninety degrees was performed, and a dorsally directed force was applied to the metacarpal head through the proximal phalanx. The wrist was then held at 10–15 degrees in extension, MCP joint from 70 to 90 degrees flexion, PIP joint from 5 to 10 degrees flexion, and DIP joint from 5 to 10 degrees flexion.

With proper cotton padding especially over the bony prominences, an ulnar gutter below-elbow plaster splint was applied. Finally, neurovascular function and capillary refill were checked. The slab was kept for 6 weeks, but after the first 3 weeks; the patients were instructed to remove the slab 3–5 times daily to allow for range of motion exercises.

### Transverse pinning (group B)

Informed consent was obtained from all cases and pre-operative labs were done. Wrist block was carried out on 36 patients and general anesthesia was given in 6 patients. All patients received a single shot of 1 gm of 3rd generation cephalosporin during induction of anesthesia and no tourniquet was applied.

**Fig. 2:**
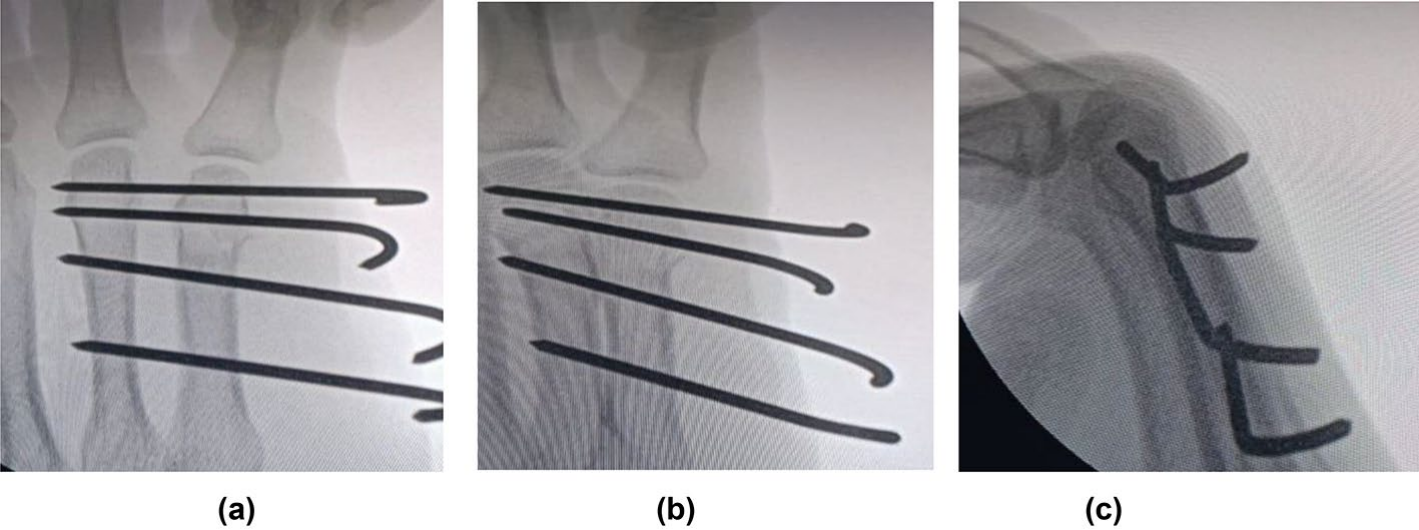
Intra-operative image intensifier photos of a patient in Group B (transverse pinning group). **a**–**c**: AP, Oblique, and lateral views respectively

All fractures were operated upon on the same day of trauma, except 2 patients who were operated upon after 5 days, since they suffered head concussion injuries that required neurosurgery observation for 48 h before surgery. First, the fracture was reduced. Then, through snip skin incisions and under image intensifier guidance, one or two transverse wires (depending on the surgeon’s preference and whether the size of the distal fragment can hold 1 or 2 wires) were inserted from the head of the fifth metacarpal to the head of the fourth metacarpal. This is followed by the insertion of two other wires from the shaft of the fifth to the shaft of the fourth metacarpal. Images were obtained in anteroposterior (AP), oblique, and lateral views at the end of the procedure to check the adequacy of reduction and fixation (Fig. 2). The wires were bent and cut prominent from the skin to facilitate their removal in the outpatient clinic at 6 weeks. Betadine-soaked gauze was wrapped around the wires. In all cases, below elbow slab extending slightly distal to the PIP was applied to help pain control usually for the first 2–3 weeks, to be followed by wrist support for another 3 weeks. The wrist support was removed 3–5 times daily to allow for gentle range of motion of the fingers and wrist.

### Follow-up measures

**Fig. 3:**
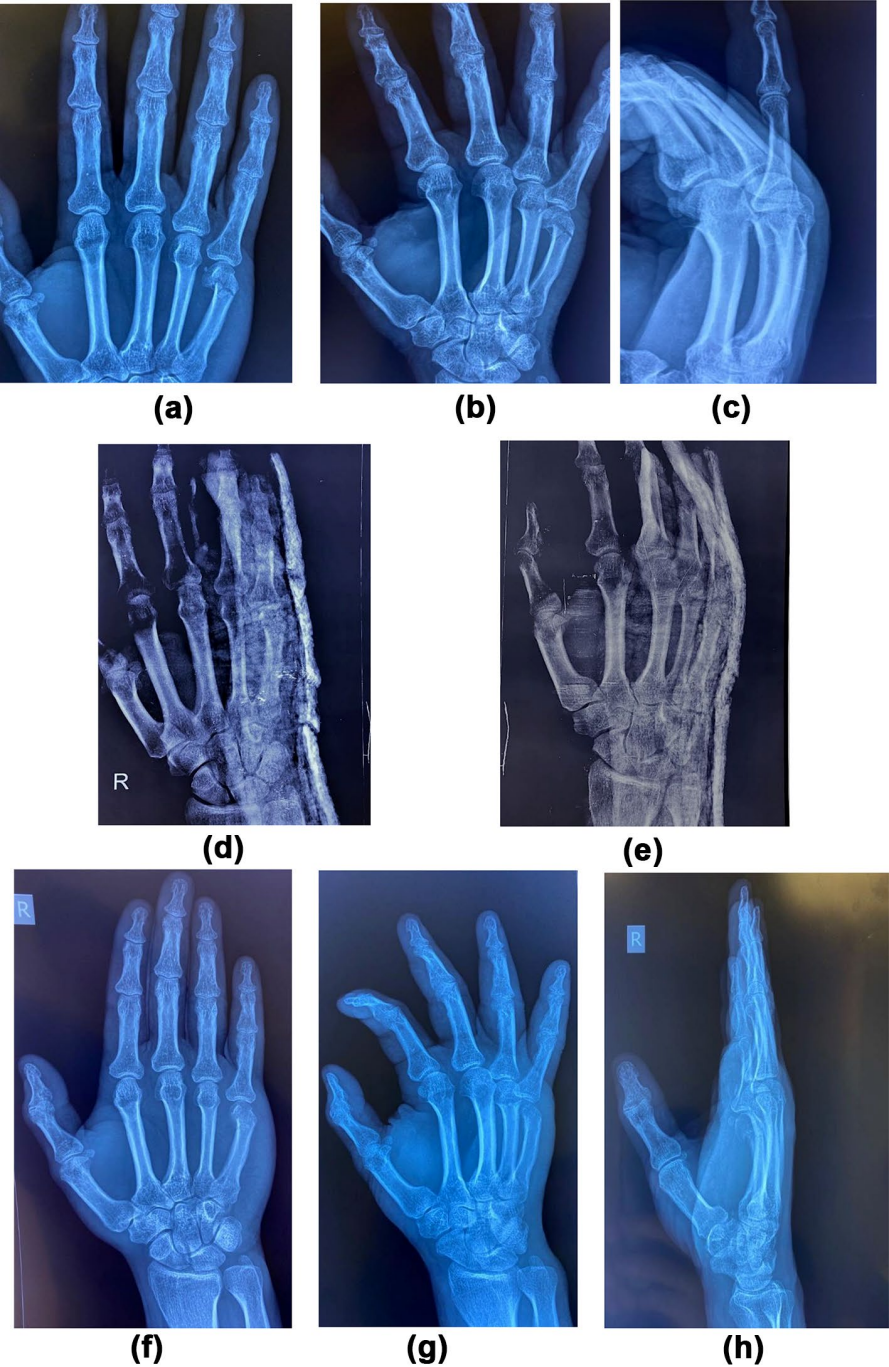
X-rays of a patient in Group A (conservative group). **a**–**c**: Pre-operative x-rays (AP, oblique, lateral views). **d**, **e**: X-rays after slab application (AP, oblique views). **f**–**h**: X-rays after union (Ap, oblique, lateral views)

**Fig. 4:**
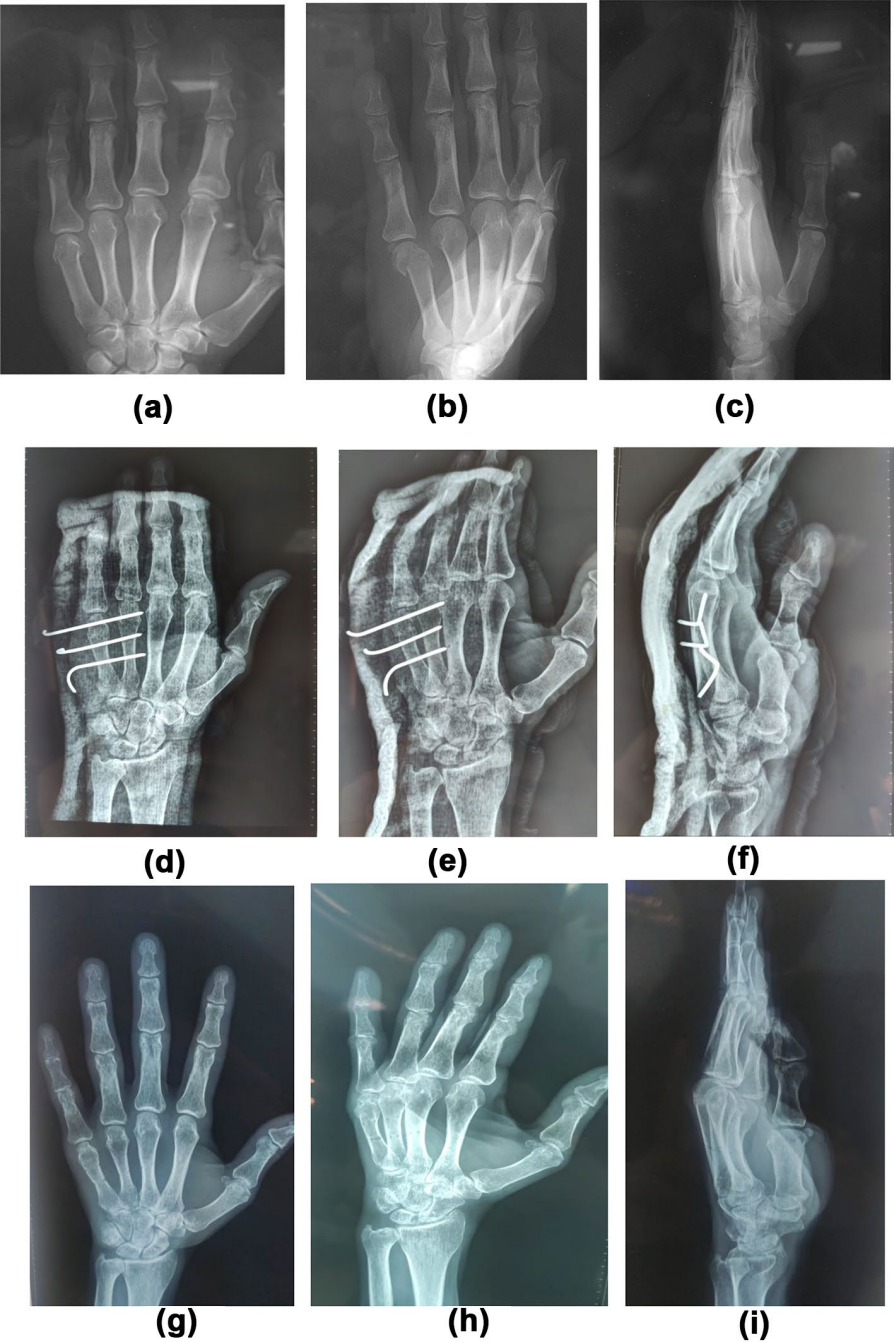
X-rays of a patient in Group B (transverse pinning group). **a**–**c**: Pre-operative x-rays (AP, oblique views, lateral). **d**–**f**: Post-operative x-rays (AP, oblique views, lateral). **g**–**i**: X-rays after union (Ap, oblique views, lateral)

**Fig. 5:**
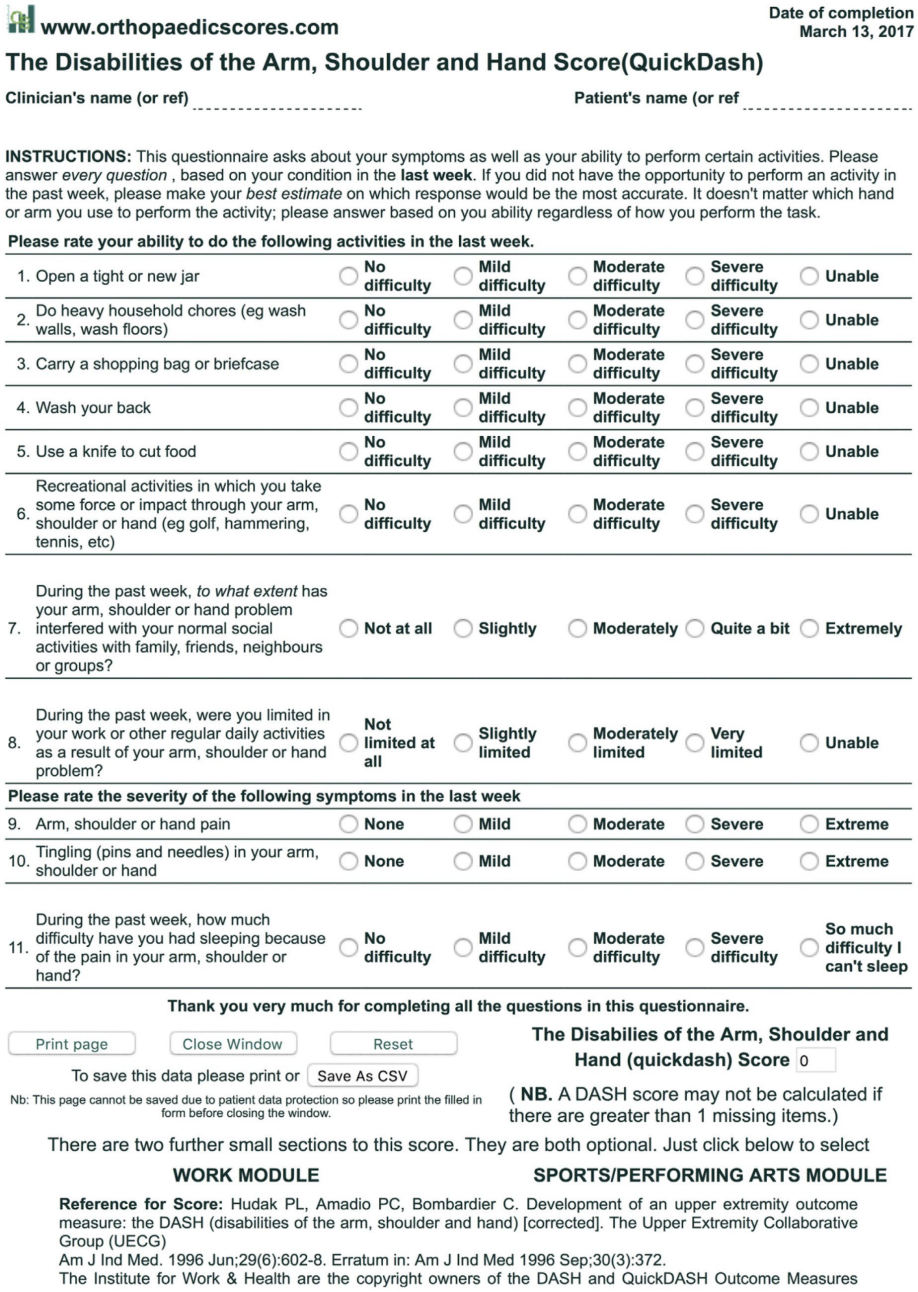
Quick DASH score

Patients were evaluated clinically and radiologically every 2–3 weeks until union, then at 6 and 12 months (Figs. 3, 4). Delayed union was considered at 3–6 months. Non-union was considered at > 6 months. At the final follow-up visit, the radiological and functional outcomes were measured and the cases were assessed using:

1. The quick DASH score (Fig. 5) [18].
2. Total active motion (TAM): this is calculated by the sum of the active range of flexion (after subtraction of the extension deficits) of the metacarpo-phalangeal (MCP), proximal inter-phalangeal (PIP) and distal inter-phalangeal (DIP) joints. Then, the score was compared to the normal range, and was given a grade accordingly [19].
3. Total Active Flexion (TAF): this is calculated by adding the sum of the active flexion range at the MCP, PIP, and DIP joints (Table 1: TAM-TAF scores) [20].

**Table 1:** American Society for Surgery of the Hand (ASSH) Total Active Motion (TAM) and Total Active Flexion (TAF) scoring systems

Total Active Motion (TAM)	
Score	TAM% (compared to contralateral finger)
Excellent	85–100%
Good	70–84%
Fair	50–69%
Poor	< 50

Total Active Flexion (TAF)	
Grade	Degree of flexion
Excellent	> 220°
Good	120–220°
Poor	< 120°

### Statistics

The data were summarized by the use of the mean and the standard deviation. For categorical data, count and percentage were used. The Mann–Whitney test was used for comparisons. A *p* value less than 0.05 was considered statistically significant. Multivariate linear regression analysis was done to detect independent predictors of full union time by entering significant variables in a stepwise linear regression model to reach a stable model for predictors in each group [21].

## Results

Ninety patients were included in this study, but only 84 cases completed the follow-up. The patients’ age ranged from 19 to 42 years. The mean age in the conservative group was 29.57 years versus 31.9 years in the transverse pinning group. No statistical significance was found between the two groups (*p* value = 0.229).

Fourteen patients were smokers in the conservative group (33.3%) versus 16 cases in the transverse pining group (38.1%). Four diabetic patients were found in each Group (9.6%), while hypertension was found in two cases in the transverse pinning group (4.8%). Four patients in the conservative group suffered from hand edema at the time of surgery versus 6 cases in the transverse pinning group (measured by the Figure-of-eight tape measure). The rest of the demographic data are summarized in Table 2.

**Table 2:** Patient Demographics and Data Analysis

	Group A Conservative		Group B Transverse Pinning		*p* value
	Number	%	Number	%	
No. of patients	42		42		
Age (years)					
Mean	29.57		31.9		0.229
Range	20–42		19–39		
Mode of trauma					
Punch	24	57.1%	30	71.4%	
Accidental fall	12	28.6%	12	28.6%	0.189
Road traffic	6	14.3%	–		
Fracture side					
Right	40	95.2%	38	90.4%	
Left	2	4.8%	4	9.6%	
Risk factors affecting the outcome					
Smoking	14	33.3%	16	38.1%	
Diabetes	4	9.6%	4	9.6%	
Hypertension	0	0	2	4.8%	
Skin condition (oedema)	4	9.5%	6	14.3%	

The average operative time in Group B was 10 (8–13) mins. The mean union time was 7.76 weeks in Group A (6–12 weeks) and 7.38 weeks in Group B (4–12 weeks) (*p* value = 0.854). Multivariate Linear regression analysis was done to detect independent predictors of Full union time. In Group A, smoking and Diabetes mellitus were found to act as significant independent predictors of full union time. In the transverse pinning Group (B), smoking, skin edema, and affection of the dominant hand (right side) constitute significant independent predictors of full union time. These factors correlated directly with the time needed to full union (Table 3). None of the cases in either group suffered from non-union.

**Table 3:** Multivariate linear regression analysis

Model	Unstandardized coefficients		Standardized coefficients	*t*	*p* value	95.0% confidence interval for B	
	*B*	Std. error	Beta			Lower bound	Upper bound
Ulnar gutter (group A)							
Full union							
(Constant)	7.192	0.030		243.222	< 0.001	7.132	7.251
Smoking	1.508	0.049	1.005	30.963	< 0.001	1.410	1.607
Diabetes mellitus	0.708	0.078	0.294	9.054	< 0.001	0.550	0.867
Transverse pinning (group B)							
Full union							
(Constant)	6.797	0.079		85.887	< 0.001	6.637	6.957
Smoking	1.140	0.047	0.947	24.165	< 0.001	1.044	1.235
Skin oedema	0.206	0.067	0.124	3.078	0.004	0.071	0.342
Affected Side = Dominant (right)	0.175	0.083	0.088	2.123	0.040	0.008	0.343

In Group A, TAM scores were excellent in 33 cases (78.6%) and good in 9 cases (21.4%). While in Group B, 35 patients (83.3%) had excellent scores versus 7 cases with good results (16.7%) (*p* = 0.08).

Total active flexion scores were excellent in 37 cases (88%) in Group A versus 39 cases (92.9%) in Group B. Five cases (12%) were good in Group A compared to 3 cases (7.1%) in Group B (*p* value = 0.872).

The mean Quick DASH score at 1 year was 2.86 in Group A, while in Group B it was 2.14. There was no statistically significant difference between the 2 groups, (*p* = 0.905). The Power of hand grip (measured by Dynamometer) as a percentage of the normal side had a mean value of 89.67 in Group A compared to 92.19 in Group B (*p* value = 0.237).

Regarding the complications, 2 cases in group B suffered from superficial infection (local redness and mild discharge at the wire entry site), which were resolved with repeated dressings. In group A, 14 patients suffered from radiographic malunion manifested as loss of the normal knuckle appearance due to palmar angulation. This had no clinical significance on the hand grip, function and the cases did not complain of cosmetic disfigurement; however, it showed statistical significance as compared to group B (*p* value < 0.05) (Tables 4, 5).

**Table 4:** Degree of Palmar Angulation

Degree of palmar angulation	Group A Conservative		Group B Transverse pinning	
	Before reduction	After reduction (bony union)	Pre-Operative	Post-operative (After union)
0–30°	–	28	–	42
30–40°	12	8	14	–
40–50°	15	6	16	–
50–60°	12	–	11	–
60–70°	3	–	1	–

**Table 5:** Functional Results

	Group A Conservative	Group B Transverse pinning	*p* value
TAM			
Excellent	33 (78.6%)	35 (83.3%)	0.08
Good	9 (21.4%)	7 (16.7%)	
TAF			
Excellent	37 (88.1%)	39 (92.9%)	0.872
Good	5 (11.9%)	3 (7.1%)	
Quick DASH Score			
Mean	2.86	2.14	0.905
Standard deviation	1.86	2.84	
Power of hand grip (% of normal side)			
Mean	89.67		0.237
Standard deviation	6.4	7.18	
Union time (weeks)			
Mean	7.76	7.38	0.854
Standard deviation	2.22	1.87	
Complications			
Superficial Infection	2 (4.7%)	–	< 0.001
Mal-union (Loss of the Knuckle)	–	14 (33.3%)	< 0.001

*TAM* Total Active Motion, *TAF* Total Active Flexion

## Discussion

The most important finding of this study is that the null hypothesis could be accepted. Conservative management of 5th metacarpal neck fractures using an ulnar gutter splint yields comparable clinical outcomes to operative intervention using transverse pinning in simple extra-articular fifth metacarpal neck fractures in active adults with palmar angulation from 30 to 70 degrees. Both methods represent effective treatment options.

Biomechanical studies suggested that malunion of fifth metacarpal neck fractures with severe palmar angulation (> 60°) and subsequent secondary shortening will result in weak fifth ray, inability to perform full clench, and finally weak hand grip [6, 7].

Up until now, there is no consensus regarding the best treatment option for this type of fracture. Recent studies are still comparing different types of conservative treatment and different surgical modalities [8–12].

In the last decade, conservative treatment has gained popularity over operative intervention. It requires less time for recovery with fewer costs. However, the main drawback is the loss of the knuckle prominence. Conservative management may be performed using POP, functional braces, splints, or even buddy tapping [9, 22, 23].

The surgical treatment options include open reduction and internal fixation using mini-plates and screws, external fixation, antegrade single or double k-wires insertion, transverse pinning, or intramedullary screw [16, 17].

Jerome et al. compared intramedullary K-wire versus conservative treatment in a POP followed by a functional splint. He found that both groups yield similar scores, but, the surgical group had better patients’ satisfaction rates and better cosmetic and functional results [12].

Antegrade wiring is the most popular surgical option in treating boxer’s fractures. It gained popularity as being minimally invasive, with very low cost, and can be performed by a single surgeon. However, the operative time is usually longer using this technique relative to other surgical options [1, 8, 14, 15].

Locked plates were used to allow for rigid fixation, early mobilization, and therefore, better union rates and less stiffness. Facca et al. compared 18 patients with boxer’s fractures treated by locked plates versus 20 patients treated by intramedullary k-wire. The results were not significant regarding functional scores, angulation, pain, strength, and time to return to work. However, the plate group showed significantly less range of motion at the metacarpophalangeal joint both in flexion and extension despite early mobilization. Furthermore, the cost and the complication rates were much higher in the plate group [13].

Only males presented with this type of fracture in our study, especially the active and productive age group as they engage in more activities and travels. This is consistent with the series of Winter et al. as boxer’s fracture is rarely met in females [8].

Regarding the fracture union, the mean time to full union showed no statistically significant difference between both studied groups. Smoking and Diabetes mellitus correlated directly with the time needed to full union in Group A, versus (smoking, skin edema, and affection of the dominant hand (right side)) in Group B. However, none of these factors resulted in non-union, as complete union was achieved in all patients in both groups.

In accordance with our results, in 2021, Bin Xu et al. studied the effect of smoking on bony union in 39,920 cases in 71 studies. They found that smokers have a higher incidence of non-union and deep wound infection when compared to non-smokers in patients with non-pathological fractures [24]. Also, Charles A. et al. in a recent study, concluded that smokers with distal upper limb fractures have a significantly higher risk of postoperative complications, un-planned reoperation, and un-planned thirty days re-admission following open reduction and internal fixation [25]. Diabetes mellitus is one of the most common and well-known factors that affect bony union. In a meta-analysis made by Zi-Chuan Ding et al., 695 diabetic cases were included and compared to 4937 controls to assess fracture union in different types of fractures including short bones. They concluded that DM significantly increased the risk of delayed fracture union with a *p* value of 0.002 [26].

Chul-Ho Kim et al. performed a multivariate analysis to study the variables affecting union in metacarpal fractures fixed by percutaneous intramedullary fixation. He included age, smoking, fracture site, body mass index, number of k-wires, and complications. They found that fractures of the shaft (versus the neck) and the greater number of k-wires used for fixation delayed the union time [27]. These results were different from our study; this may be due to the difference in the studied fracture types (Any metacarpal neck or shaft fractures). Also, age was correlated with smoking but the other factors were independent from each other.

Regarding hand dominance, no studies found a correlation between hand dominance and fracture union. However, in a study conducted by Fredrik Peyronson et al. in which conservative treatment was compared to operative management in metacarpal fractures, a linear regression analysis showed that the grip strength was better in the dominant hand by 22.2% when compared to the non-dominant side (*p* Value = 0.007). [28]

Non-union is a rare complication, as this fracture occurs in metaphyseal bone with good blood supply and healing potential. Malunion and its consequences are a concern [8, 29, 30].

When considering malunion; the conservative group (A) significantly showed higher malunion rates with loss of the normal knuckle appearance in 14 patients (33.3%) but with no effect on hand function and the patients did not complain of cosmetic disfigurement.

These results are also consistent with Westbrook et al. who compared 105 patients managed conservatively for metacarpal neck fractures and 18 patients managed operatively (using either plates or pinning). They found no significant difference regarding the functional outcomes and power grip, and that palmar angulation deformity only caused cosmetic disfigurement. This is probably because the TAF and TAM are not significantly affected by the malunion [6].

It is, however, worth mentioning that there is a huge mismatch in the number of patients in each group in Westbrook et al.’s study. Also, in the operative group they relied on two completely different techniques for fixation with different principles and therefore would lead to mixed outcomes. This represents a source of bias [6].

Regarding the operative time, in this study, the average operative time in Group B was 10 min (range 8–13 min). These were comparable to the results of Galal et al. who compared pinning to antegrade intramedullary wiring on 60 patients. In their study, the operative time in the group treated by transverse pinning was (10 ± 2 min) versus (32 ± 4 min) in the antegrade pinning group [30]. Antegrade pinning showed significantly longer operative time. Operative time varies according to the fracture pattern, degree of soft tissue edema, surgeon's experience, quality of the image intensifier, and the radiographer’s experience in providing a good view. All the patients in this study were operated upon by senior surgeons. Also, the theatre team including the radiographer are well trained to make the procedure simple and quick.

For assessment of the range of motion; two different methods were used, TAF and TAM. There was no statistically significant difference between the studied groups regarding the TAM and TAF scores. TAF is more indicative of the ability to make a clenched fist and would affect the power of the hand grip, therefore it is more useful [19, 20]. In both studied groups, TAM and TAF were either excellent or good. These results are consistent with Galal et al., who also reported no statistically significant differences among both groups with slightly better scores for the transverse pinning group as compared to antegrade pinning [30]. In the study made by Potenza et al., in which transverse pinning was performed, 26 out of 28 cases had full extension of the MCPJ. All cases achieved more than 90° flexion at the metacarpophalangeal joint and normal range of the interphalangeal joints [31]. These findings support our results that transverse pinning yields satisfactory outcomes regarding the range of motion.

When considering the complication rates in the operative group (B), two patients suffered from superficial infection, however, none suffered from mal-union. In the study done by Galal et al., postoperative complications were found only in the antegrade pinning group in which a wire backed out in one case. Two cases suffered superficial infection, and another 2 cases were complicated by injury to the dorsal sensory division of the ulnar nerve [30]. Superficial infection is a risk with pinning using K-wires especially if left protruding from the skin to facilitate later removal. Using snip skin incisions before introducing the K-wires instead of drilling directly through the skin reduces the risk of infection that may result from thermal necrosis. Patients should also be instructed on proper wire care and hygiene.

When comparing this study with the previous studies, our results, functional scores, and complication rates are comparable to other studies with different methods of fixation (Table 6-Literature review). Union rates, range of motion, and hand grip strength showed no statistically significant difference between both studied groups. Significant loss of normal knuckle appearance occurred in the conservative group and did not interfere with the hand function.

**Table 6:** Literature review findings compared with our results

	Our study	A.P. Westbrook et al. [6]	Facca et al. [13]	V. Potenza et al. [31]	S. Galal [30]
Number of Patients	84 patients	123 neck fracture patients (plates and wires) vs conservative	38 patients Plates vs Intramedullary wires	28 patients Transverse pinning	60 cases Transverse pinning vs Intramedullary wires
Mean follow up period	12 months	49 months in non-operative 42 months in operative	4.8 months in group 1 (plates) 3.3 months in group 2 (K-wires)	25 months	12 months
Mean operative time	10 min		36 min in group 1 9 min in group 2		10 min
Union	7.57 weeks			6 weeks	6–8 weeks
Functional Results	No statistical significant difference between the two groups	No statistical significant differences	No statistical significant differences But K-wires showed better mobility	Good to excellent ROM DASH score: mean value of 5	No statistical significant differences
Complications	Transverse pinning: 2 cases superficial infection	In total of 218 neck and shaft fractures: Plates: 2 sup infections 3 stiffness K-wires: 4 infections	Group I: 3 cases with limited mobility One case of head necrosis 2 cases with delayed union Group II: 3 cases of wire migration 3 neurological injury	5 cases: minor local infection	Intramedullary pinning: Wire back out:1 case -Superficial wound infection: 2 cases Injury to the dorsal branch of ulnar nerve: 2 cases

In conclusion, conservative management in ulnar gutter slab and percutaneous transverse pinning, are simple, accessible, and effective methods in the treatment of simple extra-articular fifth metacarpal neck fractures (77 A3.1 according to the AO classification) with angulation from 30 to 70 degrees. In this fracture pattern, it is recommended to counsel the patients on both methods of treatment and discuss potential knuckle loss with conservative management. Patient co-morbidities, activity level, and type should be taken into consideration. Further meta-analysis studies with different methods of fixation may be needed to support our results.

## Data Availability

Not available.

## Code availability

Not available.
